# Factors affecting wearable ECG device adoption by general practitioners for atrial fibrillation screening: cross-sectional study

**DOI:** 10.3389/fpubh.2023.1128127

**Published:** 2023-05-05

**Authors:** Yi Yao, Zhichao Li, Yi He, Yalin Zhang, Zhaoxia Guo, Yi Lei, Qian Zhao, Dongze Li, Zhi Zhang, Yonggang Zhang, Xiaoyang Liao

**Affiliations:** ^1^General Practice Ward/International Medical Center Ward, General Practice Medical Center, West China Hospital, Sichuan University, Chengdu, China; ^2^Day Surgery Center, General Practice Medical Center, West China Hospital, Sichuan University, Chengdu, China; ^3^Chengdu Seventh People Hospital, Chengdu, China; ^4^Business School, Sichuan University, Chengdu, China; ^5^Department of Emergency Medicine and Laboratory of Emergency Medicine, West China Hospital, Sichuan University, Chengdu, China; ^6^Chengdu Shuangliu District Xihanggang Community Hospital, Chengdu, China; ^7^Department of Periodical Press and National Clinical Research Center for Geriatrics, West China Hospital, Sichuan University, Chengdu, China

**Keywords:** atrial fibrillation, screening, wearable ECG device, intention, general practitioner, influencing factors, UTAUT

## Abstract

**Introduction:**

Atrial fibrillation (AF) is a challenging cardiovascular disease worldwide. Wearable electrocardiograph devices (WEDs) have great potential to improve the detection rate of AF in primary care. However, the factors that influence general practitioners’ (GPs) perception and acceptance of WEDs are not well understood. To identify factors that influence the intention of GPs to utilize WEDs in a clinical setting to screen patients for AF.

**Method:**

The research hypotheses and questionnaire items were designed and developed based on the unified theory of acceptance and technology (UTAUT) framework. We used stratified sampling and obtained the data through an online survey. Structural equation modeling was used to analyze the collected data.Results: A total of 1,004 valid questionnaires from GPs across Sichuan province in China were collected. Three factors increased GPs’ intention to utilize WEDs to screen patients for AF, including performance expectancy (*β* = 0.121, *p* = 0.004), social influence (*β* = 0.356, *p* < 0.001), and price perception (*β* = 0.587, *p* < 0.001). Perception risk (*β* = −0.059, *p* < 0.001) decreased usage intention, while effort expectancy (*β* = −0.079, *p* = 0.155) and facilitating conditions (*β* = −0.014, *p* = 0.868) did not affect usage intention. Gender (*β* = −0.022, *p* = 0.179), age (*β* = 0.006, *p* = 0.699), education level (*β* = −0.22, *p* = 0.184) and training (*β* = 0.007, *p* = 0.69) were not significantly correlated with usage intention, and these four factors had no moderating effect on the path coefficients.

**Discussion:**

GPs’ intention to utilize WEDs is affected by performance expectancy, price perception, perception risk and social influence. Researcher should improve the usability and perception of WEDs for screening and carry out studies to provide high-quality evidence for the security and efficacy of wearable devices.

## Introduction

Atrial fibrillation (AF) is the most common supraventricular arrhythmia and affects 2%–4% of the population globally (1), and exceeds 5% for those 75 years and older (2). AF is strongly associated with stroke, increasing its risk 5-fold (1). Furthermore, 38.2% of AF patients are asymptomatic, and 25% of AF diagnoses are missed because of its paroxysmal nature (3). Actively screening for AF reduced the combined endpoints of stoke, myocardial infraction, systemic embolism, and death (4). The community is a good setting for AF screening (5), and general practitioners (GPs) support for screening is available (5). The 12-lead ECG is the most common screening device in primary care. However, it is difficult to detect paroxysmal AF by 12-lead ECG because patients may be asymptomatic or have long intervals between episodes (6).

Wearable electrocardiographic devices (WEDs), which are worn on the body as an accessory to continuously collect electrocardiographic (ECG) data (7), are recommended by existing multinational AF guidelines for early screening for AF (1, 5, 8, 9). WEDs are being rapidly developed for AF screening, with more than 400 wearable monitors available, and the number is expected to double in 2021 (10–12). Moreover, WEDs increased new AF detection by 3.0% after 4 months in a randomized controlled trials (13).

**Figure 1:**
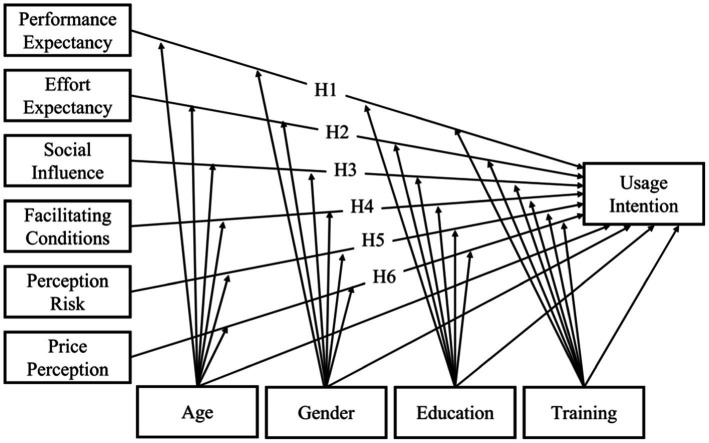
The proposed research model and hypotheses.

Unfortunately, about 1/3 of the community residents were reluctant to accept wearable devices (14, 15), and 47.1% of the users were unwilling to continually wearing the WEDs (16). In the primary care, users tend to defer their decision to GPs, which plays a pivotal role in use of internet-related technologies (17, 18). Several studies have investigated the influencing factors of users’ acceptance of WEDs but have not taken into account GPs’ intentions to recommend them (19–22). Therefore, it is necessary to identify the factors that influence GPs’ intentions to adopted WEDs (Figure 1).

### Research model and research hypotheses

The unified theory of acceptance and use of technology (UTAUT) model, which was published by Venkatesh in 2003, is recognized as a comprehensive theoretical model of usage intention (23). The UTAUT can supplement contextual constructs to adapt to different scenarios (24). We retained the four core variables in the original UTAUT model, including performance expectancy, effort expectancy, social influence and facilitating conditions (23). According to a literature review conducted by Cimperman et al. (18) and Yan et al. (25), GPs’ perceived risk may be an important factor that affects their utilization of WEDs because of the current tension between physicians and patients in China. At the same time, AF may cause a high economic burden for families (26, 27). GPs must consider the price accessibility for new healthcare equipment (1). Therefore, this study adds the variables of risk perception and price perception.

#### Performance expectancy

Performance expectancy refers to the extent to which WEDs can improve GPs’ efficiency to screen patients for AF (28). Studies showed that performance expectancy is positively correlated with physicians’ intentions to utilize WEDs for AF screening (29, 30), and decreased performance expectancy weakens the usage intention. Therefore, this paper proposes hypothesis H1:

*H1*: Performance expectancy is positively correlated with the intention to utilize WEDs for AF screening.

#### Effort expectancy

Effort expectancy refers to the degree of effort that needs to be made when adopting a new technology. Previous studies found that if learning and using a new technology is difficult, the intention to adopt the technology is weakened, and vice versa (30–32). Therefore, this paper proposes hypothesis H2:

*H2:* Effort expectancy is positively correlated with the intention to utilize WEDs.

#### Social influence

Social influence refers to the degree to which users are influenced by the people around them (33). It is generally believed that if people around a potential user are already using a product, the potential user is more willing to adopt it (34). Several studies have tested this hypothesis (29, 30, 35). For WEDs, the social influences of GPs mainly include their peers, academic guides, and media ads (TV ads or Internet ads). This article therefore proposes hypothesis H3:

*H3:* Social influence is positively correlated with the intention to utilize WEDs.

#### Facilitating conditions

Facilitating conditions refer to users’ perceptions of the degree of help provided by existing organizational and technical structures (28, 36). Studies have shown that as more facilitating conditions are established, the usage intention increases (37–39). For GPs, increasing the amount of facilitating conditions to utilize WEDs will lead to a stronger intention to utilize these devices. Therefore, this paper proposes hypothesis H4:

*H4*: Facilitating conditions are positively correlated with the intention to utilize WEDs.

#### Perception risk

In our study, perceived risk refers to an individual’s psychological perception of the risks of new technologies (40). One study found that people will not use new healthcare technology when they are skeptical about the safety of the technology (41). Other studies have reached similar conclusions (21, 42). Therefore, this paper proposes hypothesis H5:

*H5*: Perceived risk is negatively correlated with the intention to utilize WEDs.

#### Price perception

Price perception is defined as GPs’ cognitive tradeoff between the perceived benefits of the applications and the monetary cost for using new technologies (36). The price value is positive when the benefits of using a technology are perceived to be greater than the monetary cost (36). Therefore, this article proposes hypothesis H6:

*H6*: Price perception is positively correlated with the intention to utilize WEDs.

Some studies applied gender, age (28, 43, 44) and education level (44) as the moderating or controlling variables in UTAUT model. A study have shown that standardized training for residents has improved the quality of practice in China (45). GPs in China are trained in various ways (46), and training enables them to learn about new health information technologies. Thus, a GP’s training may influence his or her intention to utilize WEDs. Therefore, this study applied gender, age, education, and training as moderating and control variables. The research model is presented in Figure 1.

**Figure 4:**
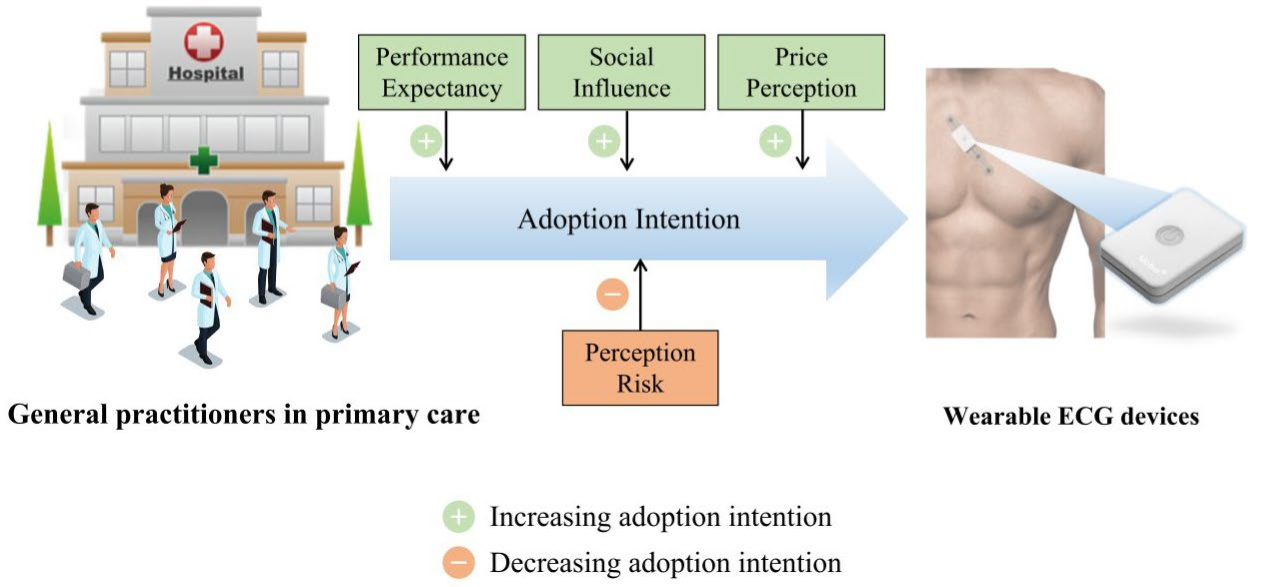
Central illustration. Factors affecting wearable ECG device adoption by general practitioners.

**Figure 2:**
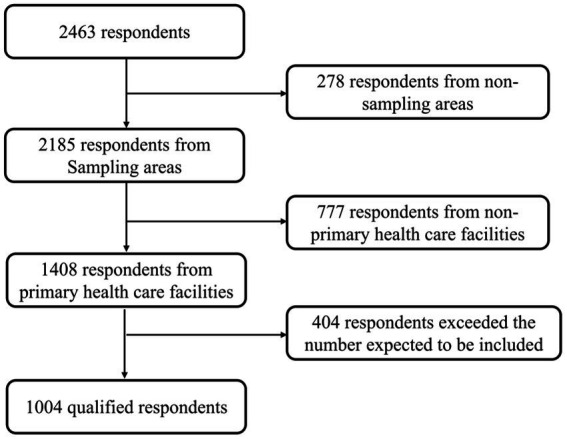
Sampling procedure. Primary healthcare facilities include community health centers and township health centers. We included questionnaires from 1,004 qualified respondents in the order we were received. The excess respondents were excluded.

**Figure 3:**
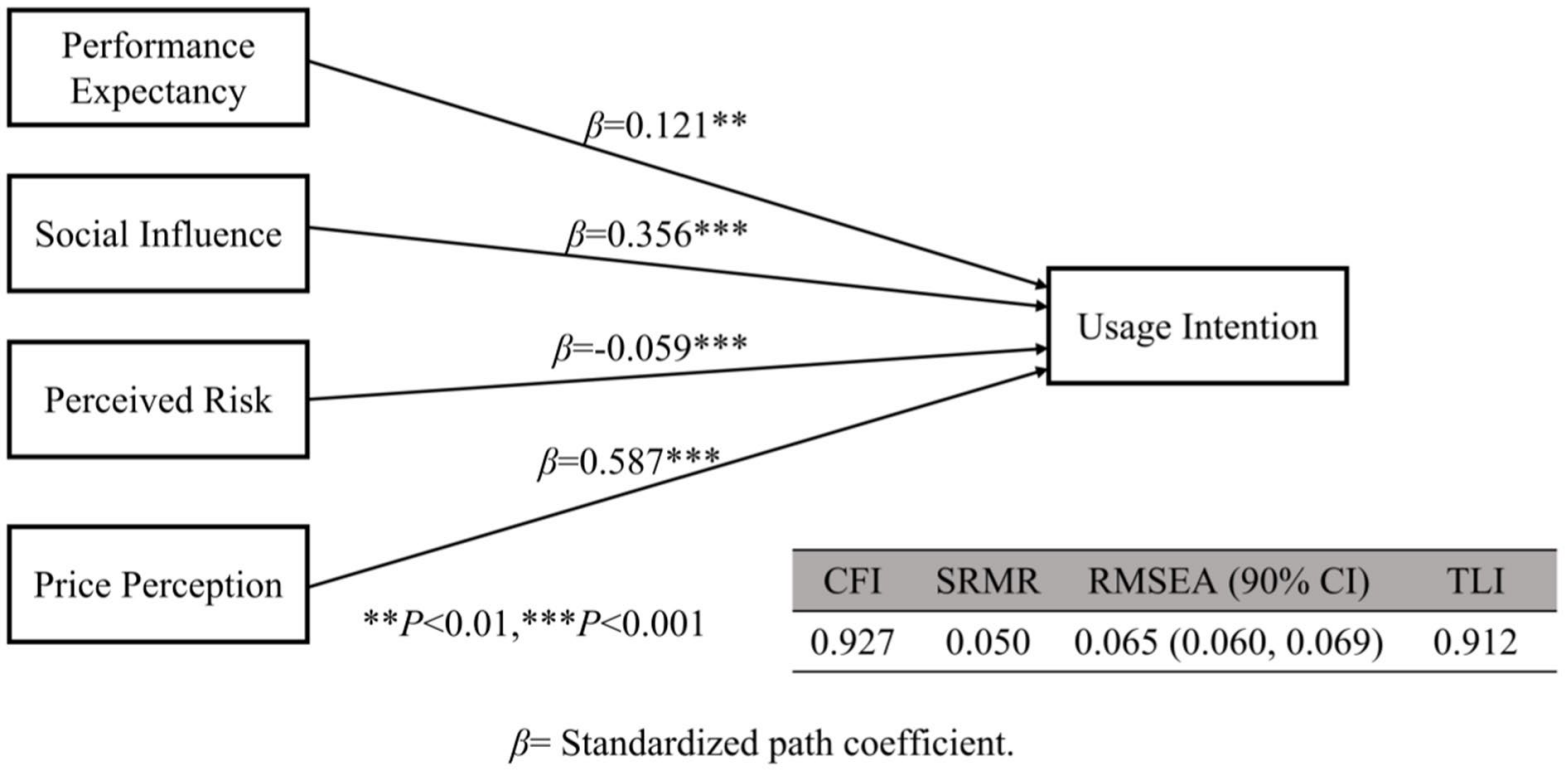
Final model.

## Methods

### Survey instrument

The survey instruments were designed after a review of the literature related to wearable technology and health information technology (18, 22, 30, 35, 43, 44, 47–57). The reviewed literature and the items of the measurement scale are detailed in Supplementary 1. First, we analyze the dimensions and the questionnaire items contained in each dimension in these literatures. Second, we determine the seven dimensions of the model in our study (Including Performance Expectancy, Effort Expectancy, Social Influence, Facilitating Conditions, Perceived Risk, Price Perception and Usage Intention). Finally, we refer to the questionnaire items in the different dimensions of above literatures. All items were translated from English to Chinese by a language expert. We then discussed all items among a panel that included a professor of general practice, a language specialist, an attending GP, and a manufacturer of WEDs. We slightly modified items to fit the scenario of WEDs and AF. The items were measured with a 5-point Likert-type scale ranging from 1 (strongly disagree) to 5 (strongly agree). The questionnaire also collected demographic information such as age, gender, education level, years working, professional title, and training. We then conducted a pilot trial with 160 participants to validate the questionnaire. See Supplementary 2 for detail. According to the pilot trial results, we made some adjustments to better fit the context. Table 1 presents the items and sources of the items that were used in the current model (not including the demographic variables).

**Table 1:** Measurement items of the constructs.

Construct	Item	Content	Sources
Performance Expectancy	PE1	It can help me obtain patient ECG data at any time.	Venkatesh (23, 36), Hailiang (22), Jewer (50), Bawack (51), Hoque (53)
	PE2	It can help me screen patients for AF.	
	PE3	It can help me diagnose AF.	
	PE4	It is helpful for my work.	
Effort Expectancy	EE1	It is easy to learn and use.	Venkatesh (23, 36), Hailiang (22), Jewer (50), Bawack (51),Hoque (53)
	EE2	It is easy to wear.	
	EE3	It is easy to operate.	
	EE4	I think it is easy to use it skillfully.	
Social Influence	SI1	It is recommended by a peer or higher-level physician, and I will use it.	Venkatesh (23, 36), Hailiang (22), Jewer (50), Bawack (51), Hoque (53)
	SI2	Media ads (TV ads or Internet ads) recommend it, and I will use it.	
	SI3	The guideline recommends it, and I will use it.	
	SI4	Other community health facilities have used it, and I will use it.	
Facilitating Conditions	FC1	My institution has the conditions to promote such equipment.	Venkatesh (23, 36), Hailiang (22), Jewer (50), Bawack (51), Hoque (53)
	FC2	I have acquired the knowledge to use this type of equipment.	
	FC3	It can complement the 24-h Holter.	
	FC4	I can get clear instructions when using it.	
	FC5	I can get the help of technicians when using it.	
Perceived Risk	PR1	I am concerned about the possible disclosure of patient privacy and health information.	Cimperman (18)
	PR2	I am worried that the results will not be accurate.	
	PR3	I am worried about patients not being able to cooperate.	
Price Perception	PP1	Its cost is reasonable.	Venkatesh (36), Garavand (44), Venkatesh (36)
	PP2	It does not increase the total cost for a patient.	
	PP3	In terms of health, it is worth it.	
Usage intention	UI1	If I had the device, I would use it in my practice.	Venkatesh (23, 36), Hailiang (22), Jewer (50), Bawack (51), Hoque (53)
	UI2	If I have patients with “palpitations,” I will have them use it.	
	UI3	If I have patients with “palpitations,” I will prioritize its usage for them.	
	UI4	I am willing to keep trying to use it for patients.	
	UI5	I would like to learn to use it in the future.	

### Participants

This cross-sectional survey was conducted among GPs in Sichuan Province. The inclusion criteria for this study were as follows: (1) a GP; (2) currently engaged in clinical-related work in primary care; and (3) provided informed consent. The exclusion criteria were as follows: (1) suffering from heart-related diseases at present or in the past; and (2) inability to use a smartphone to complete the questionnaire. The sample size is greater than or equal 200 in the statistical analysis of SEM, and should be 5–10 times the number of variables in multi-factor analysis. There were a total of 42 items in the questionnaire; therefore, 10 multiplied by 42 yields a maximum sample size of 420. The loss to follow-up was estimated at 20%, so the required sample size was estimated to be 500. This study was divided into two subgroups: 500 participants in township health centers and 500 participants in community health care centers.

To effectively evaluate factors that influence the intention of GPs to utilize WEDs, we selected Sichuan Province, which is a gateway and cultural center of Southwest China. Sichuan Province has a land area of 486,000 km^2^, ranking 5^th^ in China, and had a population of 81.40 million in 2014. There are 75,137 primary health care facilities and 4,575 township health centers in the province (58). This study used stratified sampling, applying the SPSS 23.0 random number generator for randomization to avoid selection bias. The sampling area was determined according to the classification of the medical resource allocation in Sichuan Province (59) and the classification of the five major economic zones, Supplementary 3 for details.

### Data collection

A web-based questionnaire survey *via* Sojump was applied in this study. From June 22, 2020 to July 3, 2020, we shared the survey link through a communication app (called QQ) with 1,855 GPs; QQ is a communications app that is used for academic exchange among GPs in Sichuan province. We also published the survey link on the WeChat accounts of GPs; these GPs had a total of 351 followers combined. In this way, the link to the survey was allowed to be shared through the connections of the GPs. The participants could access the questionnaire only through WeChat. Each mobile IP address could complete the questionnaire only once. Before the survey, the participants were given pictures and statements to introduce them to WEDs and AF. The picture of WEDs focused on the ECG patches, which senses the heart’s electrical signals. All participants were explicitly informed that they could complete the questionnaire anonymously and that their personal information would be strictly confidential. Informed consent was obtained from the participants before the survey was conducted. This study was approved by the Sichuan University School of Medicine Ethics Committee (Approval No. 2018–454).

### Data analysis

The data were imported into SPSS 23.0 to establish a database. We analyzed the demographic characteristics of the participants by descriptive statistics. If the measurement data conform to normal distribution, mean ± standard deviation was used; if they do not conform to normal distribution, median and interquartile range were used to describe. The Enumeration data were described by frequency and percentage. Pearson correlation coefficient was used for bivariate correlation analysis. And the bivariate correlation use the average of the values within each construct. This study used Cronbach’s α reliability coefficient to assess the reliability of the questionnaire. A Cronbach’s α coefficient > 0.9 is very credible, 0.8–0.9 is credible, 0.7–0.8 is average, 0.6–0.7 is acceptable, and below 0.6 is not credible. We also tested construct validity in this study. SPSS 23.0 software was used to calculate the Kaiser-Meyer-Olkin Measure of Sampling Adequacy (KMO) value and the Bartlett’s sphericity test value to determine whether the data were suitable for factor analysis. The standards applied were as follows: based on a Bartlett’s sphericity test result of *p* < 0.05, if the KMO value was greater than 0.9, the value was very suitable for factor analysis; 0.7–0.9 indicated the value was suitable; 0.6–0.7 indicated the value was barely suitable; and less than 0.6 indicated the value was not suitable for factor analysis. If the value was suitable for factor analysis, we used Mplus software (Version 7.4) to perform confirmatory factor analysis. The structural validity was better when the traditional factor loading cut-off values were at least 0.4. The Pearson correlation coefficient was used for bivariate correlation analysis. The larger the absolute value, the closer the relationship. A correlation coefficient above 0.7 indicated a significantly higher degree of correlation. SEM analysis was performed using Mplus statistical software (Version 7.4). The root meant square error of approximation (RMSEA), the standardized root meant squared residual (SRMR), the comparative fitting index (CFI), and the Tucker-Lewis index (TLI) were selected to evaluate the model fit. If RMSEA (90% CI) <0.08, SRMR <0.08, CFI >0.9 and TLI >0.9, we deemed the model to fit well. *p* < 0.05 (two-tailed) was considered to indicate statistical significance.

## Results

### Demographic characteristics

Figure 2 shows the sampling procedure and results. A total of 1,004 valid questionnaires were collected for this study, including 502 from township health centers and 502 from community health centers. The proportions of male and female participants were 37.6% and 62.4%, respectively. The proportions of participants aged 26–30 years old and 36–40 years old were relatively high (both close to 20%). Regarding training, only 147 participants had completed the standardized training for residents (14.6%). A total of 439 participants had undergone general practice transfer/on-the-job training sessions (43.7%). Overall, 586 participants had undergone either the standardized training for residents or general practice transfer/on-the-job training sessions (58.3%; Table 2).

**Table 2:** Characteristics of the general practitioners (*n* = 1,004).

Characteristic	Number of GPs	Percentage (%)
*Gender*		
Male	378	37.6
Female	626	62.4
*Age*		
≤25	81	8.1
26–30	193	19.2
31–35	186	18.5
36–40	199	19.8
41–45	154	15.3
46–50	118	11.8
51–55	42	4.2
>55	31	3.1
*Education level*		
Junior college and below	455	45.3
Bachelor’s degree	524	52.2
Master’s degree	24	2.4
PhD	1	0.1
*Years working*		
<5	182	18.1
5–10	264	26.3
11–20	288	28.7
>20	270	26.9
*Professional title*		
Assistant medical practitioner	290	28.9
Resident	274	27.3
Attending physician	333	33.2
Associate chief physician	95	9.5
Chief physician	12	1.2
*Type of training*		
Standardized training for residents	147	14.6
General practice transfer/on-the-job training	439	43.7
Other specialized training	121	12.1
No training	297	29.6

### Measurement model assessment

The overall Cronbach’s α reliability coefficient of the questionnaire in this study was 0.957, and the Cronbach’s α reliability coefficients of all questionnaire dimensions were greater than 0.8, indicating high internal consistency. The KMO value for this questionnaire was 0.963 (>0.7), and Bartlett’s sphericity test gave a chi-square value of 26801.13 (*p* < 0.01). In the confirmatory factor analysis, all factor loadings ranged from 0.596 to 0.951; they were all greater than 0.4, which met the traditional factor loading cut-off value, demonstrating good structural validity (Table 3).

**Table 3:** Cronbach’s *α* coefficients, confirmatory factors of the questionnaire.

Construct	Item	Mean score (SD)	Confirmatory factor	Cronbach’s α
Performance expectancy	PE1	4.41 (0.70)	0.861	0.935
	PE2	4.43 (0.69)	0.934	
	PE3	4.35 (0.77)	0.887	
	PE4	4.41 (0.68)	0.872	
Effort expectancy	EE1	4.19 (0.78)	0.865	0.948
	EE2	4.25 (0.77)	0.880	
	EE3	4.19 (0.77)	0.951	
	EE4	4.15 (0.80)	0.927	
Social influence	SI1	4.30 (0.70)	0.860	0.856
	SI2	3.76 (1.02)	0.682	
	SI3	4.34 (0.70)	0.817	
	SI4	4.20 (0.73)	0.826	
Facilitating conditions	FC1	3.98 (0.93)	0.686	0.853
	FC2	3.47 (1.14)	0.596	
	FC3	4.15 (0.76)	0.827	
	FC4	4.10 (0.77)	0.902	
	FC5	4.12 (0.76)	0.851	
Perceived risk	PR1	3.26 (1.18)	0.778	0.856
	PR2	3.26 (1.10)	0.895	
	PR3	3.50 (1.04)	0.895	
Price perception	PP1	3.69 (0.83)	0.769	0.848
	PP2	3.60 (0.91)	0.750	
	PP3	4.05 (0.77)	0.750	
Usage intention	UI1	4.15 (0.72)	0.899	0.946
	UI2	4.17 (0.71)	0.907	
	UI3	4.09 (0.78)	0.889	
	UI4	4.14 (0.72)	0.856	
	UI5	4.25 (0.66)	0.867	

SD, Standard deviation.

Six latent variables were found to be significantly correlated with usage intention: effort expectancy (*r* = 0.712, *p* < 0.01), social influence (*r* = 0.774, *p* < 0.01), facilitating conditions (*r* = 0.755, *p* < 0.01), and price perception (*r* = 0.736, *p* < 0.01) had high, positive correlations with usage intention, while performance expectancy (*r* = 0.681, *p* < 0.01) had a moderate, positive correlation with usage intention. Perceived risk (*r* = 0.146, *p* < 0.01) had a low, positive correlation with usage intention. There were significant correlations among all of the six variables, except for between perceived risk and performance expectancy (Table 4).

**Table 4:** Correlation analysis of influencing factors and usage intention (*n* = 1,004).

	Performance expectancy	Effort expectancy	Social influence	Facilitating conditions	Perceived risk	Price perception	Usage intention
Performance expectancy	1						
Effort expectancy	0.678**	1					
Social influence	0.686**	0.777**	1				
Facilitating conditions	0.599**	0.736**	0.776**	1			
Perceived risk	0.057	0.122**	0.139**	0.241**	1		
Price perception	0.505**	0.611**	0.662**	0.749**	0.314**	1	
Usage intention	0.681**	0.712**	0.774**	0.755**	0.146**	0.736**	1

**p < 0.01 indicates the correlation is significant.

### Structural equation model testing

Performance expectancy (*β* = 0.199), social influence (*β* = 0.403), and price perception (*β* = 0.585) had significant positive effects on usage intention (*p* < 0.01). Perceived risk had a significant negative effect on usage intention (*β* = −0.085). The effects of effort expectancy and facilitating conditions on usage intention were not statistically significant (Table 5).

**Table 5:** Results of path analysis and hypothesis testing.

Hypothesis	Pathway	*β*	SE	*p* value	Result
1	Performance Expectancy → Usage Intention	0.199	0.039	0.002	Supported
2	Effort Expectancy → Usage Intention	−0.079	0.055	0.155	Not supported
3	Social Influence → Usage Intention	0.403	0.082	<0.0001	Supported
4	Facilitating Conditions → Usage Intention	−0.014	0.087	0.868	Not supported
5	Perceived Risk → Usage Intention	−0.085	0.019	<0.0001	Supported
6	Price Perception → Usage Intention	0.585	0.105	<0.0001	Supported

β, Standardized path coefficient; SE, Estimated standard error.

After setting gender, age, education level and training as regulatory variables, they were found education level have influence on the path coefficient from social influence to usage intention (*p* = 0.023). Other regulatory variables have no influence on the path coefficient from each variable to usage intention (*p* > 0.05). Furthermore, gender, age, education level and training did not influence usage intention (*p* > 0.05) (Tables 6, 7).

**Table 6:** Effect of regulating variables on the path coefficient.

Pathway	Age		Gender		Education level		Training	
	*β*	*p* value	*β*	*p* value	*β*	*p* value	*β*	*p* value
Performance Expectancy→ Usage Intention	−0.017	0.827	0.075	0.144	−0.163	0.058	0.034	0.687
Effort Expectancy→ Usage Intention	0.063	0.485	−0.091	0.091	−0.111	0.225	−0.082	0.412
Social Influence→ Usage Intention	−0.038	0.791	0.075	0.400	0.385	0.023	−0.055	0.732
Facilitating Conditions→ Usage Intention	−0.146	0.341	−0.005	0.953	−0.231	0.125	0.149	0.331
Perceived Risk→ Usage Intention	0.000	0.999	−0.002	0.883	0.032	0.157	−0.001	0.980
Price Perception→ Usage Intention	0.149	0.305	−0.048	0.553	0.143	0.298	−0.104	0.448

β, Standardized path coefficient.

**Table 7:** Degree of influence of control variables on usage intention in the model.

Control variable	*β*	*p* value
Age	−0.022	0.179
Gender	0.006	0.699
Education level	−0.220	0.184
Training	−0.007	0.690

β, Standardized path coefficient.

According to the hypothesis testing of this model, effort expectancy and facilitating conditions did not affect usage intention; therefore, we deleted them. Gender, age, education level and training had no statistically significant impact on the usage intention. Therefore, control variables were not added to this model. Only education level have influence on the path coefficient from social influence to usage intention. We added education level as regulated variables for social influence to usage intention. We obtained a new model based on that analysis. We found that education level have no influence on the path coefficient from social influence to usage intention (*p* = 0.210) in new model. So we deleted the regulated variable “education level.” And we obtained a final model with a CFI of 0.927, an SRMR of 0.050, an RMSEA (90% CI) of 0.065 (0.060, 0.069), and a TLI of 0.912. In the final model, the path coefficients of performance expectancy, social impact, perceived risk, and price perception were 0.121, 0.356, −0.059, and 0.587, respectively, and all *p* values were less than 0.01 (Figure 3).

## Discussion

This study adopted the UTAUT to explore the adoption of WEDs to screen patients for AF among GPs in Sichuan Province of China. Performance expectancy, social influence, and price perception increased the GPs’ intention to utilize WEDs, while perceived risk decreased usage intention. Effort expectancy and facilitating conditions did not affect usage intention.

For GPs who stated that they would utilize WEDs to screen for AF, the more effective the devices, the stronger the willingness to adopt them. This finding was consistent with previous studies (22, 52, 56). Performance expectancy was an indispensable independent variable in the UTAUT model. Considered to be the most important influencing factor for usage intention in previous studies (53, 60, 61). A qualitative study by Volpato et al. showed GPs expressed a desire to become more involved in the development of wearables technologies because it play an increasingly central role in daily practices (62). But health professionals still had concerns about efficacy in illness and disease prevention for its use (63). The performance expectations were based on the GP’s belief that the equipment was reliable. Therefore, randomized controlled trials are required to provide higher quality evidence for the reliability and usefulness of WEDs.

In addition, social influence was positively affected the willingness of GPs to utilize WEDs in their practice. This finding was consistent with the original assumptions of the UTAUT model and previous studies (22, 64). In our study, the more recommended these devices were by the social environment (e.g., guidelines, peers), the stronger the practitioners’ intention was to use them. And the social influences that affect practitioners’ use of WEDs were mainly their peers, higher-level physicians, and academic guidelines. However, different guidelines had different recommend for using wearable ECG devices. For example, the European Society of Cardiology (ESC) had recommended wearable technology in AF screening (65), but the National Institute for Health and Care Excellence (NICE) did not (66). Undoubtedly, there are many challenges and barriers to adoption of WEDs for practitioners and rigorous studies are warranted (67). It will be beneficial to adoption and promotion if the researchers can provide high-quality evidence for diagnostic performance.

If GPs were skeptical about the safety of WEDs, these devices would be rejected. Here, the perceived risk had a negative impact on the intention to use WEDs. The higher the perceived risk is, the lower the intention to use the device. This is consistent with previous results (64). Drehlich et al. conducted semi-institutional interviews with 144 adolescents and found that perceived risk was an important factor affecting adolescents’ acceptance of the combination of wearable pedometers and Facebook (68). How to ensure the data security while using wearable devices is a problem worth studying (69). In our study, many GPs were concerned that the device could leak patients’ private health information. Therefore, manufacturers of WEDs should strengthen the protection of user information (62, 70, 71).

A better price perception was related to a stronger willingness to utilize WEDs to screen patients for AF. GPs in China were required to provide basic medical services for residents in the area under their jurisdiction (72). Often, local medical institutions could not carry out expensive auxiliary examinations due to limited resources. As long as the equipment was of sufficient quality, GPs hoped that the devices would be inexpensive, thus enabling WEDs to become more accessible to people.

If a device was simple to use, people would be more likely to use it. However, in this study, there was no significant correlation between effort expectancy and usage intention. This was inconsistent with the original assumptions of the UTAUT model and also in contrast to most previous research results (22, 24). The reasons might be as follows. The GPs were intellectuals who received good information technology education (73), and they believed that WEDs were easy to operate. In their questionnaire responses, the GPs stated that operating and wearing the WEDs was simple and convenient and that it was easy to learn and use these devices. Therefore, the influence of effort expectancy on the intention to use was not a driving factor. The higher the degree of facilitating conditions, the stronger the usage intention. The results of the model showed that the facilitating conditions had no effect on usage intention, which was inconsistent with most findings (20, 22). The reason might be that, in general, medical resources were insufficient in primary care, and most of the GPs worked under conditions that were unable to promote the use of these devices. Finding ways to improve the facilitating conditions of local medical institutions was worth further discussion.

## Contributions and implications

This study confirmed the significant roles of performance expectancy, social influence, price perception and perceived risk in predicting the intention of GPs to utilize WEDs in their practice. To our knowledge, this was the first study to specifically investigate the intention of GPs to utilize wearable devices. Previous studies on people’s willingness to use wearable technology mostly focused on specific consumer groups, such as older adults, women, teenagers, and patients with diabetes (21, 22, 29, 42, 68, 74, 75). Our study provided a preliminary foundation for the selection and continuous use of these devices in primary care. It used stratified sampling in Sichuan Province, located in southwest China. This sampling strategy was an improvement over previously used simple sampling methods. Our sample population was more representative than in previous studies and avoided selection bias, to some extent. Furthermore, the sample size was larger than in previous studies. We collected 1,004 valid questionnaires in multiple prefectures, and effectively avoided the problem of a small sample size. Finally, the structural equation model was used in our study; this analysis method has advantages over the multiple linear regression method of Macdonald’s study (75).

## Limitations

There were limitations in this study. First, it investigated only the GPs’ intention to use WEDs without further researching actual usage behaviors. The intention to use may not completely represent actual usage. Usage behavior could be investigated and explored to determine various latent variables and their influence on usage intention and actual usage behavior. Second, although it was based on the UTAUT model, with perceived risk and price perception added to clarify the direct impacts on usage intention, it explored only four moderating variables: gender, age, education level, and general training. In real life, usage might be affected by other variables, such as personal cognition, attitudes, and behaviors. Thus, the model could be further improved in the future. Third, the study is based mainly on GPs from Sichuan Province and the applicability of these results with different demographics should be investigated further. In addition, this study examined the willingness of GPs who worked in primary healthcare facilities; practitioners who work in tertiary general hospitals should also be investigated. Fourth, each of the constructs of “usage intention” get at different aspects of use. “I would like to learn it in the future” is much more hypothetical and future. “I am willing to keep trying to use it for patients.” is much more concrete and current. It is necessary to consider looking into relationships between the constructs and each individual usage question in future.

## Conclusion

Based on the UTAUT model, this study constructed a model for predicting GPs’ willingness to adopt WEDs to screen patients for AF. Performance expectancy, social influence, and price perception positively affected the willingness of GPs to utilize WEDs in their practice. Perceived risk negatively affected the willingness to utilize WEDs (Figure 4). Effort expectancy and facilitating conditions did not affect usage intention in this research model. Therefore, researchers should improve the usability of WEDs, and carry out study to provide high-quality evidence for safety and efficacy of these devices, which will benefit their promotion. The theoretical and practical implications were provided for GPs to increase use of WEDs in their healthcare activities.

## Data availability statement

The raw data supporting the conclusions of this article will be made available by the authors, without undue reservation.

## Ethics statement

The studies involving human participants were reviewed and approved by the Human Ethics Committee of West China Hospital of Sichuan University. The patients/participants provided their written informed consent to participate in this study.

## Author contributions

YY, ZL, YH, YZ, and XL conceived and planned the study. ZG performed the analysis. YY, ZL, and DL wrote the original draft with input from all authors. QZ, YL, and YLZ visualized the results. ZG, ZZ, and YL contributed to the interpretation of the results. ZG, YZ, and XL critically revised the article. YZ and XL supervised the entire process. All authors contributed to the article and approved the submitted version.

## Funding

This work was supported financially by the Science and Technology Department of Sichuan Province (2020YFSY0014), the Sichuan Province Health Research Project (19PJ094), and the Sichuan Medical Association (HX-H1910211).

## Conflict of interest

The authors declare that the research was conducted in the absence of any commercial or financial relationships that could be construed as a potential conflict of interest.

## Publisher’s note

All claims expressed in this article are solely those of the authors and do not necessarily represent those of their affiliated organizations, or those of the publisher, the editors and the reviewers. Any product that may be evaluated in this article, or claim that may be made by its manufacturer, is not guaranteed or endorsed by the publisher.
