# Supplementary material for: Factors affecting wearable ECG device adoption by general practitioners for atrial fibrillation screening: cross-sectional study
Source: Front Public Health. 2023 May 5;11:1128127. doi: 10.3389/fpubh.2023.1128127 (PMC10196261; doi:10.3389/fpubh.2023.1128127)
Supplement: Supplementary file 1 [file Table_1.docx]

# *Supplementary Material*

**Factors Affecting** **Wearable ECG Device Adoption by** **General Practitioners for Atrial Fibrillation Screening: Cross-Sectional Study**

**Yi Yao^1†^,Zhichao Li^2†^,** **Yi He^1,3^,** **Yalin Zhang^1^, Zhaoxia Guo^4^, Yi Lei^1^, Qian Zhao^1^, Dongze Li^5^, Zhi Zhang^6^, Yonggang Zhang^7^*, Xiaoyang Liao^1^***

^1^ General Practice Ward/International Medical Center Ward, General Practice Medical Center, West China Hospital, Sichuan University, Chengdu, China.

^2^Day Surgery Center, General Practice Medical Center, West China Hospital, Sichuan University, Chengdu, China.

^3^Chengdu Seventh People Hospital, Chengdu,China.

^4^ Business School, Sichuan University, Chengdu, China.

^5^Department of Emergency Medicine and Laboratory of Emergency Medicine, West China Hospital, Sichuan University, Chengdu, China.

^6^ Chengdu Shuangliu District Xihanggang Community Hospital, Chengdu, China.

^7^ Department of Periodical Press and National Clinical Research Center for Geriatrics, West China Hospital, Sichuan University, Chengdu, China

^†^These authors have contributed equally to this work and share first authorship.

***Corresponding authors**

Xiaoyang Liao

Email: [liaoxiaoyang@wchscu.cn](mailto:liaoxiaoyang@wchscu.cn).

Yonggang Zhang

Email: jebm_zhang@yahoo.com.

# Supplementary 1 The validated measurement scales of reference to the reviewed literature.

**Supplementary 1 Table 1 Details about the** **validated measurement scales** **of reference to the reviewed literature**

| Authors | Publication Time | Research object | Core variables | Moderating or controlling variables |
| --- | --- | --- | --- | --- |
| Wang H et al.(1) | 2020 | Healthcare Wearable Devices | Social Influence, Facilitating Conditions, Effort Expectancy, Performance Expectancy, Behavioral Intention, Technology Characteristics, Task Characteristics, Task-Technology Fit | - |
| Petersen F et al.(2) | 2020 | Mobile Health  Applications for Diabetic | Performance Expectancy, Effort Expectancy, Social Influence, Facilitating Conditions, Behavioral Intention, Use Behavioral | Gender, Age, Experience, Voluntariness of Use |
| Pichandy C et al.(3) | 2020 | Information and Communication Technology | Performance Expectancy, Effort Expectancy, Social Influence, Facilitating Condition, Behavioural Intention, Use Behavior | Age, Gender, Experience,  Voluntariness of Use |
| Shiferaw K B et al.(4) | 2019 | Electronic Medical Record System | Performance Expectancy , Effort Expectancy , Social Influence , Facilitating Conditions | - |
| Garavand , A et al.(5) | 2019 | Mobile Health | Performance Expectancy , Effort Expectancy , Social Influence , Facilitating Conditions, Behavioral Intention, Use Behavioral | Gender, Age, Educational Degree |
| Lu, X et al.(6) | 2019 | Online Health  Communities | Performance Expectancy , Effort Expectancy , Social Influence, Attitude toward Using Technology, Behavioral Intention | Gender, Age, Living Area, Educational Degree |
| Magsamen-  Conrad K et al.(7) | 2019 | New Communication Technology | performance expectancy, effort expectancy, social  influence, facilitating conditions, and intention; attitudes,  anxiety  performance expectancy, effort expectancy, social  influence, facilitating conditions, and intention; attitudes,  anxiety  performance expectancy, effort expectancy, social  influence, facilitating conditions, and intention; attitudes,  anxiety  performance expectancy, effort expectancy, social  influence, facilitating conditions, and intention; attitudes,  anxiety  performance expectancy, effort expectancy, social  influence, facilitating conditions, and intention; attitudes,  anxiety  performance expectancy, effort expectancy, social  influence, facilitating conditions, and intention; attitudes,  anxiety  performance expectancy, effort expectancy, social  influence, facilitating conditions, and intention; attitudes,  anxiety  performance expectancy, effort expectancy, social  influence, facilitating conditions, and intention; attitudes,  anxiety  performance expectancy, effort expectancy, social  influence, facilitating conditions, and intention; attitudes,  anxiety  performance expectancy, effort expectancy, social  influence, facilitating conditions, and intention; attitudes,  anxiety  performance expectancy, effort expectancy, social  influence, facilitating conditions, and intention; attitudes,  anxiety  Performance Expectancy , Effort Expectancy , Social influence, Intention, Attitudes, Anxiety | Age, Sex, Experience, Level of Education |
| Hossain A et al.(8) | 2019 | Electronic Health Record | Performance Expectancy , Effort Expectancy , Social Influence, Facilitating Conditions, Behavioral Intention, Use Behavior, Personal Innovativeness in IT, Resistance to Change | - |
| Jewer, J (9) | 2018 | An Emergency Department Wait-times Website, | Performance Expectancy , Effort Expectancy , Social Influence, Facilitating Conditions, Behavioral Intention, Use | Gender, Age, Experience |
| Bawack, R E et al.(10) | 2018 | Health Information Systems | Performance Expectancy , Effort Expectancy , Social Influence, Self-efficacy, Cost-effectiveness, Facilitating Conditions, Behavioral Intention | Gender, Age, Experience, Voluntariness of Use |
| Quaosar G et al.(11) | 2018 | Mobile-health Services | Performance Expectancy , Social Influence , Effort Expectancy, Facilitating Condition, Perceived Credibility, Behavioral Intention | - |
| Hoque, R et.al(12) | 2017 | Mobile Health | Performance Expectancy , Effort Expectancy, Social Influence , Facilitating Condition, Behavioral Intention, Use behavior, Technology Anxiety, Resistance to Change | - |
| Kim, S et al.(13) | 2016 | Mobile Electronic Medical Record | Performance Expectancy , Effort Expectancy, Social Influence, Attitude, Facilitating Condition, Intention to Use | - |
| Cimperman, M et al(14) | 2016 | Home Telehealth Services | Performance Expectancy, Effort Expectancy, Facilitating Conditions, Social Influence, Doctor’s Opinion, Computer Anxiety, Perceived Security, Behavioral Intention to Use | - |
| Vanneste D et al.(15) | 2013 | A Web-based System Enabling Person-centred Recording and Data Sharing across Care Settings | Performance Expectancy, Effort Expectancy, Social Influence, Facilitating Conditions, Anxiety, Self-efficacy, Attitude towards Using Technology, Behavioural Intention | - |
| Kijsanayotin B et al.(16) | 2009 | Health Information Technology | Performance Expectancy, Effort Expectancy, Social Influence, Voluntariness, Intention to Use, Facilitating Conditions, IT Experience, IT Knowledge, Care and Report Use, Administration Use | - |
| El-Gayar O F et al(17) | 2008 | Electronic Medical Records | Performance Expectancy, Effort Expectancy, Facilitating Conditions, Social Influence, Behavioral Intention, Use | - |

IT: information technology

# Reference

1. Wang H, Tao D, Yu N, Qu X. Understanding consumer acceptance of healthcare wearable devices: An integrated model of UTAUT and TTF. *Int J Med Inform* (2020) 139:104156. Epub 2020/05/11. doi: 10.1016/j.ijmedinf.2020.104156.

2. Petersen F, Jacobs M, Pather S. Barriers for User Acceptance of Mobile Health Applications for Diabetic Patients: Applying the UTAUT Model. (2020) 12067:61-72. doi: 10.1007/978-3-030-45002-1_6.

3. Pichandy C, Rathinaswamy J, koothoor P. Technology Acceptance By Medical Doctors In India: An Analysis With UTAUT Model. *International Journal of Scientific & Technology Research* (2020) 9:3854-7.

4. Shiferaw KB, Mehari EA. Modeling predictors of acceptance and use of electronic medical record system in a resource limited setting: Using modified UTAUT model. *Informatics in Medicine Unlocked* (2019) 17:100182. doi: 10.1016/j.imu.2019.100182.

5. Garavand A, Samadbeik M, Nadri H, Rahimi B, Asadi H. Effective Factors in Adoption of Mobile Health Applications between Medical Sciences Students Using the UTAUT Model. *Methods Inf Med* (2019) 58(5):131-9. doi: 10.1055/s-0040-1701607.

6. Lu X, Zhang R, Zhu X. An Empirical Study on Patients' Acceptance of Physician-Patient Interaction in Online Health Communities. *Int J Environ Res Public Health* (2019) 16(24). Epub 2019/12/18. doi: 10.3390/ijerph16245084.

7. Magsamen-Conrad K, Wang F, Tetteh D, Lee YI. Using Technology Adoption Theory and a Lifespan Approach to Develop a Theoretical Framework for eHealth Literacy: Extending UTAUT. *Health Commun* (2019):1-12. Epub 2019/07/23. doi: 10.1080/10410236.2019.1641395.

8. Hossain A, Quaresma R, Rahman H. Investigating factors influencing the physicians’ adoption of electronic health record (EHR) in healthcare system of Bangladesh: An empirical study. *International Journal of Information Management* (2019) 44:76-87. doi: <https://doi.org/10.1016/j.ijinfomgt.2018.09.016>.

9. Jewer J. Patients' intention to use online postings of ED wait times: A modified UTAUT model. *Int J Med Inform* (2018) 112:34-9. Epub 2018/03/04. doi: 10.1016/j.ijmedinf.2018.01.008.

10. Bawack RE, Kala Kamdjoug JR. Adequacy of UTAUT in clinician adoption of health information systems in developing countries: The case of Cameroon. *Int J Med Inform* (2018) 109:15-22. Epub 2017/12/03. doi: 10.1016/j.ijmedinf.2017.10.016.

11. Quaosar G, Hoque MR, Bao Y. Investigating Factors Affecting Elderly's Intention to Use m-Health Services: An Empirical Study. *Telemed J E Health* (2018) 24(4):309-14. Epub 2017/10/05. doi: 10.1089/tmj.2017.0111.

12. Hoque R, Sorwar G. Understanding factors influencing the adoption of mHealth by the elderly: An extension of the UTAUT model. *Int J Med Inform* (2017) 101:75-84. Epub 2017/03/30. doi: 10.1016/j.ijmedinf.2017.02.002.

13. Kim S, Lee KH, Hwang H, Yoo S. Analysis of the factors influencing healthcare professionals' adoption of mobile electronic medical record (EMR) using the unified theory of acceptance and use of technology (UTAUT) in a tertiary hospital. *BMC Med Inform Decis Mak* (2016) 16:12. Epub 2016/02/03. doi: 10.1186/s12911-016-0249-8.

14. Cimperman M, Makovec Brenčič M, Trkman P. Analyzing older users' home telehealth services acceptance behavior-applying an Extended UTAUT model. *Int J Med Inform* (2016) 90:22-31. Epub 2016/04/23. doi: 10.1016/j.ijmedinf.2016.03.002.

15. Vanneste D, Vermeulen B, Declercq A. Healthcare professionals' acceptance of BelRAI, a web-based system enabling person-centred recording and data sharing across care settings with interRAI instruments: a UTAUT analysis. *BMC Med Inform Decis Mak* (2013) 13:129. Epub 2013/11/28. doi: 10.1186/1472-6947-13-129.

16. Kijsanayotin B, Pannarunothai S, Speedie SM. Factors influencing health information technology adoption in Thailand's community health centers: applying the UTAUT model. *Int J Med Inform* (2009) 78(6):404-16. Epub 2009/02/07. doi: 10.1016/j.ijmedinf.2008.12.005.

17. El-Gayar OF, Wills M, Bennett D. Examining healthcare professionals’ acceptance of electronic medical records using UTAUT. *issues in information systems* (2008) 9(2):396-401.

# Supplementary 2 The pilot trial with 160 participants.

Before the formal issuance of the questionnaire, we conducted pilot trial in 160 participants. the Cronbach's α reliability coefficients of all questionnaire dimensions were greater than 0.8, indicating high internal consistency. The KMO value for this questionnaire was 0.925 (>0.7), and Bartlett's sphericity test gave a chi-square value of 3923.057 (*P*<0.01). (Supplementary 2 Table 1 and Supplementary 2 Table 2)

**Supplementary 2 Table 1 Cronbach's α coefficients of the questionnaire in pilot trial**

| Construct | Cronbach's α | Number of items |
| --- | --- | --- |
| Performance Expectancy | 0.910 | 4.00 |
| Effort Expectancy | 0.930 | 4.00 |
| Social Influence | 0.856 | 4.00 |
| Facilitating Conditions | 0.820 | 5.00 |
| Perceived Risk | 0.804 | 3.00 |
| Price Perception | 0.830 | 3.00 |
| Usage Intention | 0.917 | 5.00 |

**Supplementary 2 Table 2 KMO and Bartlett's sphericity test**

| KMO value | | 0.925 |
| --- | --- | --- |
| Bartlett's sphericity test | A chi-square value | 3923.057 |
|  | Degree of freedom | 378.000 |
|  | Prominence | 0.000 |

#

# Supplementary 3 The sampling procedure.

The allocation of medical resources in Sichuan Province was divided into three categories:Type 1 regions (regions with sufficient resources), Type 2 regions (regions with medium resources), and Type 3 regions (regions with insufficient resources). The five major economic zones in Sichuan are determined by the Sichuan provincial government and include the plain economic region of Chengdu, the economic zone of southern Sichuan, the economic zone of northeastern Sichuan, the Panxi economic zone, and the economic zone of northwestern Sichuan. A total of 8 regions were identified (Supplementary 3 Table 1).

**Supplementary 3 Table 1 Five major** **economic zones and** **medical resource allocation in Sichuan Province, China**

| Region | Plain economic region of Chengdu | Economic zone of southern Sichuan | Panxi economic zone | Economic zone of northeastern Sichuan | Economic zone of northwestern Sichuan |
| --- | --- | --- | --- | --- | --- |
| Type 1 | Chengdu |  | Panzhihua |  |  |
| Type 2 | 1 Deyang  2 Mianyang  3 Meishan  4 Ziyang | 1 Zigong  2 Luzhou  3 Yibin  4 Neijiang  5 Leshan | Ya'an | 1 Suining  2 Nanchong  3 Dazhou  4 Guang'an  5 Bazhong  6 Guangyuan |  |
| Type 3 |  |  | Liangshan Prefecture |  | 1 Aba Prefecture  2 Ganzi Prefecture |

The numbers indicate the sample code.

Among the above eight regions, the Chengdu, Panzhihua, Ya'an, and Liangshan Prefectures are located in only one region, so these prefectures were directly included in the survey. The remaining 4 regions were numbered separately and were randomly sampled. The selected regions were the Meishan, Zigong, Dazhou, and Aba Prefectures. The sampling results are shown in Supplementary 3 Table 2.

**Supplementary 3 Table 2 Sampling results**

| Region | Plain economic region of Chengdu | Economic zone of southern Sichuan | Panxi economic zone | Economic zone of northeastern Sichuan | Economic zone of northwestern Sichuan |
| --- | --- | --- | --- | --- | --- |
| Type 1 | Chengdu |  | Panzihua |  |  |
| Type 2 | Meishan | Zigong | Ya'an | Dazhou |  |
| Type 3 |  |  | Liangshan Prefecture |  | Aba Prefecture |

The weight of the percentage of physicians in each region was calculated according to the number of physicians working in community health centers and township health centers in the "*Sichuan Provincial Health Statistics Yearbook 2018*". The percentage was multiplied by the total sample size of 500 to obtain the sample size for each city. We used the rounding method in the process of calculating the sample size. The sample sizes of community health care centers were as follows: 379 in Chengdu, 29 in Panzhihua, 15 in Meishan, 22 in Zigong, 7 in Ya'an, 29 in Dazhou, 18 in Liangshan, and 3 in Aba. The sample sizes of the township hospitals were as follows: 189 in Chengdu, 14 in Panzhihua, 58 in Meishan, 44 in Zigong, 28 in Ya'an, 100 in Dazhou, 57 in Liangshan and 12 in Aba (Supplementary 3 Table 3). A total of 1,004 practitioners (community health centers: 502, township health centers: 502) were needed to participate in the study.

**Supplementary 3 Table 3 Sample distribution by region**

| Region | Community Health Centers | | |  | Township Health Centers | | |
| --- | --- | --- | --- | --- | --- | --- | --- |
|  | Number of medical practitioners | Proportion (%) | Practitioner sample size |  | Number of medical practitioners | Proportion (%) | Practitioner sample size |
| Chengdu | 2774 | 75.7 | 379 |  | 2822 | 37.8 | 189 |
| Panzhihua | 214 | 5.8 | 29 |  | 210 | 2.8 | 14 |
| Meishan | 106 | 2.9 | 15 |  | 867 | 11.6 | 58 |
| Zigong | 160 | 4.4 | 22 |  | 648 | 8.7 | 44 |
| Ya'an | 50 | 1.4 | 7 |  | 410 | 5.5 | 28 |
| Dazhou | 211 | 5.8 | 29 |  | 1491 | 19.9 | 100 |
| Liangshan | 131 | 3.6 | 18 |  | 848 | 11.3 | 57 |
| Aba | 20 | 0.5 | 3 |  | 179 | 2.4 | 12 |
